# Does the SCL-90-R predict weight loss 12 months post-Roux-en-Y gastric bypass? Letter to the Editor (Research letter, in response and addition to the article by Albert et al. 2022)

**DOI:** 10.1007/s40519-023-01637-1

**Published:** 2024-01-25

**Authors:** Niel Merckx, Laurence Claes, Maud De Venter, Philip Plaeke, Anthony Beunis, Martin Ruppert, Guy Hubens, Filip Van Den Eede

**Affiliations:** 1 Psychiatric Hospital Multiversum, Campus Broeders Alexianen, Provinciesteenweg 408, 2530 Boechout, Belgium; 2 Faculty of Medicine and Health Sciences, Collaborative Antwerp Psychiatric Research Institute (CAPRI), University of Antwerp (UA), Universiteitsplein 1, 2610 Antwerp, Belgium; 3 Faculty of Psychology and Educational Sciences, University of Leuven (KU Leuven), Tiensestraat 102, PO box 3720, 3000 Leuven, Belgium; 4 ‘t Kader vzwResidentiële Verslavingszorg, Ericastraat 15, 2440 Geel, Belgium; 5 Department of Abdominal Surgery, Antwerp University Hospital (UZA), Wilrijkstraat 10, 2650 Edegem, Belgium; 6 Antwerp Surgical Training, Anatomy and Research Centre (ASTARC), Faculty of Medicine and Health Sciences, University of Antwerp (UA), Universiteitsplein 1, 2610 Antwerp, Belgium; 7 Department of Psychiatry, Antwerp University Hospital (UZA), Wilrijkstraat 10, Europe, 2650 Edegem (Antwerp), Belgium

**Keywords:** Anxiety, Bariatric surgery, Gastric bypass, Morbid obesity, Outcome predictors, Psychological screening, Weight loss

Dear Editor,

We have read the article by Albert et al. with a lot of interest and we would like to report on the following study and results, which are partly in line with the findings of Albert et al. [1].

In Belgium, preoperative psychological screening has become the standard procedure for bariatric surgery. However, the exact psychological predictors of (un)successful surgery in terms of weight loss still need to be refined. The aim of the current study was to evaluate the capacity of the SCL-90-R to predict weight loss following Roux-en-Y gastric bypass (GB), hypothesising that higher scores predict less weight loss.

Candidates for GB to be performed in the Antwerp University Hospital completed the Dutch version of the SCL-90-R before surgery [2]. In a retrospective and observational cohort design, we included consecutive patients from a systematic clinical database for whom sufficient follow-up data were available. The study was approved by the ethics committees of the Antwerp University Hospital and the University of Antwerp in 2018 (file code 18/01/004) and was conducted in accordance with the declaration of Helsinki.

We adopted hierarchical linear regression analyses to assess the predictive value of the SCL-90-R total score and subscale scores, always controlling for the following three variables: age, sex/gender identity, and preoperative body mass index (step 1). The primary analysis included the scale’s total score as the main predictor of weight loss in terms of the percentage of total weight lost at 12 months post-GB (%TWL12; dependent variable); the secondary analyses included the nine subscale scores. The independent variable was deemed predictive if it had a significant beta coefficient and both the explanatory variance and significance of the model increased in step 2. A *p* value of < 0.05 was considered statistically significant. According to G*Power, a sample size of 43 achieves 80% power to detect a correlation of at least 0.20 at a significance level of 0.05 with four predictors.

Of the 144 patients screened, the data of 65 (45%) were retained for analysis. Reasons for exclusion were insufficient follow-up data, having undergone a gastric-sleeve procedure instead of GB, having had prior bariatric surgery, the SCL-90-R being completed postoperatively only, cancellation of GB or conversion to open surgery. The mean age of the patients included was 43 years (*SD* = 13; range 18 to 68) and the mean BMI before surgery 42.36 (*SD* = 4.47; range 35.00 to 55.11); 44 (68%) were women and 21 (32%) men. The use of psychopharmacological agents showed no significant correlations with weight loss (*r* = − 0.10; *p* = 0.41).

The results of the significant hierarchical linear regression analyses can be found in Table 1. In the primary analysis, the SCL-90-R total score barely failed to reach significance (*β* = − 0.230; *p* = 0.067) as a predictor for the %TWL12. In the secondary analysis, we only found the anxiety subscale to significantly negatively predict %TWL12 (*β* = − 0.279; *p* = 0.025), although this finding did not withstand correction for multiple testing (10 comparisons).

**Table 1:** Hierarchical linear regressions

		*β*	*p* value	*R*^2^	Adjusted *R*^2^	*p* value
Step 1	BMI	0.073	0.690	0.111	0.067	0.065
	Gender	0.010	0.936			
	Age	− 0.158	0.019			
Step 2a	BMI	0.059	0.637	0.160	0.104	0.031
	Gender	0.067	0.597			
	Age	− 0.326	0.013			
	SCL-90 tot	− 0.230	0.067			
Step 1	BMI	0.073	0.690	0.111	0.067	0.065
	Gender	0.010	0.936			
	Age	− 0.158	0.019			
Step 2b	BMI	0.178	0.470	0.183	0.128	0.015
	Gender	0.079	0.532			
	Age	− 0.291	0.024			
	Anxiety	− 0.279	0.025			

*%TWL12* percentage total weight loss at 12 months, *BMI* body mass index, *SCL-90 tot* SCL-90-R total score; *β* standardised beta coefficient, *Anxiety* SCL-90-R Anxiety subscale

Contrary to our initial assumption, the SCL-90-R total score did not significantly predict %TWL12. Notably, the original SCL-90 scales did not predict weight loss in bariatric patients in the larger study (*N* = 334) by Albert et al. [1] either. However, after assigning the 90 items of the SCL-90 to eight factors, the factor analysis demonstrated that the empirical factors “relational distress” and “anxiety” did significantly predict post-surgery weight loss. Thus, combined with our finding, anxiety may still be an interesting factor to assess pre- and post-bariatric surgery and to further examine in future studies.

Our study suffers from the limitation that a number of variables that may moderate the association between SCL-90-R scores and the outcomes such as the effects of pre- and postoperative support and patient care protocols were not considered. Also, the reasons for dropout during follow-up were not always (clearly) documented, where it cannot be ruled out that in some patients their dropping out may have been mediated by anxiety issues. Last, and inherent to self-reporting, there is the risk of socially desirable responding, which has been shown to occur in preoperative work-ups [3].

In summary, we can conclude that the SCL-90-R total score has little predictive value for weight loss following gastric-bypass surgery. Scores on its anxiety subscale do reflect that higher preoperative symptoms of anxiety are associated with reduced weight loss following GB, although this preliminary finding requires further research in a larger sample.
